# Supplementary material for: Process evaluation of a hybrid effectiveness-implementation, pragmatic, cluster randomised controlled trial (IMPULSE) to improve psychosocial treatment of patients with psychotic-spectrum disorders in Southeast Europe
Source: PLoS One. 2026 Feb 4;21(2):e0338408. doi: 10.1371/journal.pone.0338408 (PMC12872023; doi:10.1371/journal.pone.0338408)
Supplement: S1 File — List of questions asked during interviews with patients and clinicians. (DOCX) [file pone.0338408.s001.docx]

**Supporting File 1.** Interview topic guides with clinicians and patients from the intervention arm of the IMPULSE trial.

**Experiences of DIALOG+ intervention
[patient participants]**

*Topic guide for interviews with patients who used the DIALOG+ intervention*

**BACKGROUND (3 min)**

Ask participant to introduce themselves and say a little bit about themselves. Interviewer to begin.

**TO EXPLORE PATIENT’S EXPERIENCE OF USING DIALOG+ (20 min)**

1. For the last year or so, as part of this study, you started using a tablet to discuss a set of topics during your meetings with your clinician. This intervention is called DIALOG+. **Can you talk me through how you and your clinician would use this and what happened during the sessions?**

***Probes:***

- *How easy or difficult did you find to participate in the DIALOG+ sessions? What made it easy/difficult?*
  - *Did your mental of physical health prevent you from attending/participating the sessions in any way?*
  - *How easy or difficult did you find using the tablet during sessions?*
  - *Did DIALOG+ cause you any discomfort?*
  - *If you had difficulties with participating in DIALOG+, how did you overcome them?*
- *How much did you like or dislike conversations you had in DIALOG+ sessions?*
- *Were DIALOG+ sessions too short or too long for you?*
- What did you think about having 6 DIALOG+ sessions over 12 months? Would you have liked to do it more or less often?
- *How confident did you feel about participating in DIALOG+ sessions?*
- *Were family members/carers involved in your DIALOG+ sessions? Was this helpful/useful?*
- *Considering your responses, overall, how appropriate did you feel these DIALOG+ sessions were for you?*

1. **How did your experience change over time? (compare 5-6^th^ session with 1-2 session, change for the better or worse)**

**TO EXPLORE THE IMPACT OF DIALOG+ ON PATIENTS LIFE (5 min)**

1. **How do you think participating in the DIALOG+ sessions has affected you?**

***Probes:***

- *How has your overall situation changed, if at all? In what ways has it improved/not improved?*
- How helpful/useful was participating in the DIALOG+ sessions?
- *Did family members/carers notice any change?*

**TO EXPLORE THE COLLABORATIVE WAY OF WORKING BETWEEN PATIENTS AND CLINICIANS (10 min)**

1. The DIALOG+ sessions may have been different from the usual meetings with your clinician/psychiatrist. **What was the same and what was different for you, compared to your meetings in the past?**

***Probes:***

- *Were you asked to choose topics for discussion in DIALOG+ sessions?*
- *Who would decide on activities/homework, you or/and your clinicians? How did you feel about that?*
- *Would you have liked the topics and/or activities to be chosen in a different way? (e.g. you alone, clinician alone or a joint decision)*
- *Do you think you were seen as a partner in these discussions?*

1. At the end of each session, you and your clinician set actions/activities/homework to be completed before the next session. **What did you like or dislike about setting and completing actions?**

***Probes:***

- *What was easy or difficult about setting the actions?*
- *Do you feel you were able to complete the actions set before your next DIALOG+ session? What was easy or difficult about completing the actions?*
- *Did your family members support you in completing actions? In what way?*
- *Did you use the patient booklet in setting and completing actions? Did you find it useful? Why/why not?*
- *Did you and your clinician talk about the actions from the previous session at the beginning of the next session?*
- *What actions, if any, were important to you?*

**TO EXPLORE SCALING UP AND SUSTAINABILITY OF DIALOG+ (5 min)**

1. **Would you like to continue using the DIALOG+ intervention? Why/why not?**

***Probes:***

- *Do you think the DIALOG+ intervention would be useful for other patients? Why/why not?*
- *Do you think that the DIALOG+ intervention should be offered to patients as part of the clinical care in this country? Why/why not? If yes, how can that happen*

**TO EXPLORE SUGGESTED IMPROVEMENTS OF USING DIALOG+ (5 min)**So far we discussed DIALOG+ or the intervention that uses the tablet, which you had a chance to try out. At the end let’s speak about what needs to be improved.

1. **If we were to offer this intervention to other patients, could you please suggest what would we need to improve so that other patients could also successfully use it?**

**CLOSING REMARKS (5 min)**

Do you have anything else to add to what we have discussed today: any further comments or recommendations?

**Experiences of DIALOG+ intervention
[clinician participants]**

*Topic guide for interviews with clinicians who delivered the DIALOG+ intervention*

**BACKGROUND (3 min)**

Ask the participant to introduce themselves and say a little bit about themselves.

**TO ASSESS THE FIDELITY OF THE INTERVENTION DELIVERY (20 -25 min)**

1. For the last year or so you have been delivering the DIALOG+ intervention to patients. **Can you talk me through how you and your patients would use DIALOG+ and what happened during the sessions?**

***Probes:***

- *How easy or difficult did you find it to deliver the DIALOG+ intervention? What made it easy/difficult? Is there anything that would make it easier?*
  - *Please consider what was easy or difficult in regards to patients, clinicians and services involved in the intervention?*
  - *Were there any patients that DIALOG+ did not work well with?*
  - *What do you think of the training and supervision you received from researchers during the study?*
  - *What kind of support did you have from your service manager/line manager?*
  - *If you had difficulties, how did you overcome them?*
- *How did you find conversations you had with your patients in DIALOG+ sessions?*
- *How did you manage the time it took to deliver DIALOG+? Were DIALOG + sessions too short or too long?*
- *How did you find using a tablet computer in your work with patients?*
- *How confident did you feel about delivering DIALOG+?*
- How did your experience change over time?

**TO EXPOLORE PERCEIVED USEFULNESS/EFFECTIVENESS (15 - 17 min)**

1. **Do you think there have been benefits, if any, to the use of the DIALOG+ intervention? If yes, what were they and how were they achieved?**

***Probes:***

- - *What changes have you noticed in your patients’ behaviour and communication? What do you think has caused these changes?*
  - *Have your practice or service improved in any way?*
  - *Considering your responses, overall, how appropriate do you think these DIALOG+ sessions were for your practice?*
  - *What did not work well in the Dialog+ intervention?*

**TO EXPLORE THE COLLABORATIVE WAY OF WORKING BETWEEN PATIENTS AND CLINICIANS (20 min)**

1. The DIALOG+ sessions may have been different from your usual routine meetings with patients. **How did the DIALOG+ approach change your communication with your patients included in the intervention arm of the trial?**

***Probes:***

- *How did using a tablet and DIALOG+ impact your therapeutic relationship?*
- *Do you think your patients became more proactive in discussions?*
  - *Were DIALOG+ sessions more patient-led as compared to before?*

1. At the end of each session, you and your patients set actions/activities/homework to be completed before the next session. **How did you go about setting actions?**

***Probes:***

- *Did you review action items from a previous session?*
- *Do you feel that the actions agreed were mutually agreed between you and the patients?*
- *What was easy/difficult about setting the actions?*
  - *What would make it easier to set actions? Is there anything that helped you?*

**TO EXPLORE THE SUSTAINABILITY OF THE INTERVENTION (10 min)**

1. **Would you like to continue using DIALOG+ as part of your routine clinical practice? Why/why not?**

***Probes:***

- *Do you think that the DIALOG+ intervention should become part of the clinical practice in this country?*
- *What do you think needs to change in services (service provision, clinicians) for the DIALOG+ intervention to become part of the clinical practice?*

1. **If you had to suggest just one key improvement for other clinicians to successfully deliver DIALOG+ in their routine clinical meetings what would you say**?

**CLOSING REMARKS (5 min)**

Do you have anything else to add to what we have discussed today: any further comments or recommendations?
