# Supplementary material for: Process evaluation of a hybrid effectiveness-implementation, pragmatic, cluster randomised controlled trial (IMPULSE) to improve psychosocial treatment of patients with psychotic-spectrum disorders in Southeast Europe
Source: PLoS One. 2026 Feb 4;21(2):e0338408. doi: 10.1371/journal.pone.0338408 (PMC12872023; doi:10.1371/journal.pone.0338408)
Supplement: S1 Table — Summary of results, divided into barriers, facilitators, and triangulation, from three data sources per each contextual attribute. (DOCX) [file pone.0338408.s002.docx]

| **Supporting Table 2.** Findings from data analysis and triangulation of pre-trial focus groups (General FGs), initial site visits and mental health policy analysis per each contextual attribute from Squires et al. (2019). | | | | |
| --- | --- | --- | --- | --- |
| **Contextual Attribute & Definition** | **Summary of findings (Barriers)** | **Summary of findings (Facilitators)** | **Triangulation** (per contextual feature) | |
| **1) Resource Access** |  |  |  | |
| *Access to resources of whatever kind.* | - Participants reported that there is lack of time available during routine clinical meetings, linked to the large volume of patients. - The number of clinicians in mental health facilities was interpreted as low and insufficient given the volume of patients (2.68 - 9.98 psychiatrists per 100,000 inhabitants; 21-35 mental health professionals per 100,000 inhabitants). - It was reported that the mental health facilities do not have tablets. Not all facilities have Wi-Fi. - Clinicians reported limited space to hold patient meetings. | - Resources were perceived as sufficient by some. Some patients perceived no lack of space within mental health facilities. There was space allocated at the mental health facilities for DIALOG+ implementation during the trial. - The short length of clinical meetings was perceived as suboptimal. - Likert scales were reported to be used in the treatment process during routine clinical meeting for documentation. Additionally, a more practical, structured and visual way to track patient's progress over time and compare current clinical meetings with previous ones was perceived as missing. - Policymakers perceive nurses as suitable to deliver a psychosocial intervention. - Mental health facilities are in the process of modernization. - Type of treatments offered include: pharmacotherapy & various psychological interventions (family interventions, occupational therapy, psychoeducation, CBT, art therapy, psychotherapy). Wider social aspects of patients' lives were reported to be discussed during regular therapeutic sessions. It was reported that a model of coordinated care & principles of CBT are followed, and that setting tasks between clinical meetings and assessing patient’s quality of life is part of the clinical work in the mental health services. However, it was also reported that the existing treatment for patients mostly follows a medical model, where routine clinical meetings are focused on patients' medications and care is not patient-centred nor focused on patient’s quality of life, which are aspects that were viewed as valuable to patients' care. | **Time as a resource:** Silence (General FGs) **Documentation:** Silence (General FGs) **Staff:** Partial agreement (Site Visits, Policy Analysis & General FGs) - some participants in the General FGs perceived the number of staff to be sufficient **Programs:** Agreement (General FGs, Site Visits, Policy Analysis) | |
| **2) Work Structure** |  |  |  | |
| *The arrangement of tasks, responsibilities, and resources within and between the various teams working in a clinical setting.* |  | - Routine outpatient meetings last between 15-45 min. Clinical meeting are not perceived as standardized between different clinicians nor that they follow a particular structure. - Routine outpatient meetings take place from once a month to once every 6 months if the patient is stable. - Improved system for coordination and continuity of care was reported as needed. | **Timeframe:** Silence (Site Visits) **Continuity of Care:** Silence (General FGs) **Standardization of Care:** Silence (General FGs) **Work Tempo:** Silence (Site Visits) | |
| **3) Financial** |  |  |  | |
| *Monetary receipts (income) and expenditures (costs) relating to clinical behaviour or institutional standards.* | - Mental health facilities were reported as being funded based on the number of patients seen doing clinical meetings, no incentives are included for the quality of services provided. Clinicians reported that utilizing other mental health professionals besides psychiatrists in mental health service delivery would require changes to the funding system of mental health care. Doctors were perceived as being underpaid. - Specialized therapies are perceived as expensive for the available financial resources. Clinicians report that institutions lack technological equipment and financial resources to procure it. - The cost of travel to mental health facilities is reported as expensive for patients. | - Psychological interventions provided in all state health institutions are free of charge and are covered by the national health insurance funds. | **Costs:** Silence (General FGs) **Funding System:** Silence (Policy Analyses & General FGs) **Financial Incentives:** Silence (General FGs) | |
| **4) Patient Characteristics** |  |  |  | |
| *The attributes of individuals under medical care or treatment.* | - Cognitive skills of some patients were perceived as low. | - Clinicians perceived patients as able to actively participate in clinical meetings, describe their physical and mental wellbeing & come up with tasks that would help them in their recovery. Patients were perceived as capable to gain skills by learning from their clinicians. - It was reported that patients can choose the clinician they want to talk to during routine clinical meetings. - Some patients reported being technology savvy, whereas others would need to receive training if some technology is to be used in their clinical meetings. - Patients see the inclusion of carers/family members in their care as important. | **Demographics:** Silence (General FGs) **Patient Expectations and Preferences:** Silence (General FGs) | |
| **5) Facility Characteristics** |  |  |  | |
| *The attributes of a building or group of buildings designated as a site for providing healthcare. These characteristics include the type of facility (i.e. a hospital, a walk-in clinic, a trauma centre, etc.); the volume of patients cared for at that location; the geographic location; the geographic catchment; and the presence or absence of medical trainees.* | - There is a lack of CMHCs in Kosovo* (n=8, staff is lacking), North Macedonia (n=7, located in cities), Montenegro (n=7, located in bigger cities) & Serbia (n=1, CMHCs do not exist as regular services in the mental healthcare system). The outpatient mental health care is hospital-based in Bosnia and Herzegovina, Serbia, North Macedonia and Montenegro. - Hospital-based mental health care remains dominant and there are a large number of hospitals specializing in treatment for psychiatric disorders. In Bosnia and Herzegovina there are four main psychiatric hospitals. In Kosovo* there is the Psychiatric clinic in Pristina and regional psychiatric wards. In North Macedonia there are three psychiatric hospitals. In Montenegro there are two main psychiatric hospitals and there are departments of psychiatry in all general hospitals in the country. In Serbia there are ten psychiatric hospitals. - The volume of patients cared for in the mental health facilities was reported as high: Bosnia and Herzegovina (8716 patients, 48% with psychosis); Kosovo*(8,919 patients, 2,022; 22.7% with psychosis); North Macedonia (26,288 sessions, 60-70% with psychosis); Montenegro (14,000 caseloads, 50-60% with psychosis); Serbia (34,000 outpatient visits, no % reported with psychosis). - Mental health facilities are located mostly in urban areas, and cover both rural and urban populations. | - All countries have some established CMHCs that contribute to the process of deinstitutionalization in Southeast European countries. Bosnia & Herzegovina has a large network of CMHCs (n=72, covering both urban and rural areas with approximately 66.000 inhabitants). The outpatient mental health care is community-based in Kosovo*. - A positive atmosphere that is relaxed and accepting during clinical meetings is valued. - Caregivers viewed the mental health facilities as old-fashioned. | **Type of Facility:** Silence (Site Visits) **Geography:** Silence (Policy Analysis) **Volume:** Silence (General FGs & Site Visits) **Atmosphere:** Silence (General FGs) **Facility Characteristics (general):** Silence (Policy Analysis) | |
| **6) Professional Role** |  |  |  | |
| *A set of expectations, both formal and informal, associated with a given clinical occupation.* | - Clinicians' technical skills were perceived as limited, as well as skills in psychotherapy. Caregivers expressed doubt that clinicians are familiar with a solution-focused treatment approach. | - Doctors are trained in psychological interventions. - It was reported that the mental health staff is highly motivated to strengthen the outpatient community services. | **Clinical Skill Set:** Silence (General FGs) **Professional Role Training:** Silence (General FGs) **Job Autonomy:** Silence (Site Visits) | |
| **7) Culture** |  |  |  | |
| *The inherited ideas, beliefs, values, and attitudes of a group.* |  | - Institutional support/approval was seen as vital for innovation implementation in the clinical services. The management of the health facilities was perceived to influence the willingness of clinicians to receive training in a novel intervention (hierarchical management). - Caregivers valued patient-centred care and active patient involvement in clinical meetings. Clinicians valued participation of caregivers in the patient's care. Involving more non-psychiatrists in mental health care was reported as important. | **Organizational Culture:** Silence (General FGs) | |
| **8) Health Care Professional Characteristics** |  |  |  | |
| *The attributes of individuals working as providers of medical care.* |  | - It was reported that clinicians have experience with using technology & psychosocial approaches in their clinical work. | **Experience:** Silence (General FGs) | |
| **9) Collaboration** |  |  |  | |
| *To work jointly with others (including other organizations) or together especially in an intellectual endeavour.* |  | - It was reported that there is a small number of mental health clinicians, enabling a strong network of friendship and collaboration. | **Social Interactions:** Silence (General FGs) | |
| **10) Evaluation** |  |  |  | |
| *The systematic collection of information about the activities, characteristics, and outcomes of programs, services, policies, or processes, in order to make judgments about the program/process, improve effectiveness, and/or inform decisions about future development.* | No data coded to this attribute. | No data coded to this attribute. | No data coded to this attribute. | |
| **11) System Features** |  |  |  | |
| *Distinct characteristics of a group of related parts that move or work together in order for a health care region, organization, hospital or clinical practice to run effectively.* | - Most mental health facilities in all 5 countries still rely to some extent on hard copies of patient records. - There is a lack of service user/citizens organizations that support patients and their families in Kosovo* (n~1), North Macedonia (n~1), Montenegro (n~0) & Serbia (n~2). | - Clinicians take notes during and after clinical meetings in ways that are perceived as inefficient use of time that could be instead directed to better care. - Mental health facilities in all countries have established health information systems and electronic patient records. All countries maintain medical records in both analog and digital form. - NGOs and service user/citizens organizations were identified in all countries, expect in Montenegro, and they support people with mental illness with various activities focused on adapting to the social environment, self-acceptance and self-expression. Bosnia & Herzegovina has a large network of service user organizations (n~15) that support patients and their families in collaboration with Clinical Centre and CMHCs. | **Resource Waste:** Silence (General FGs) **Record-Keeping:** Agreement & Silence (Site Visits & Policy Analysis) **Logistic and coordination:** Silence (Policy Analysis) | |
| **12) Societal Influences** |  |  |  | |
| *The general level of social knowledge and attitude as it regards to a particular clinical behaviour or procedure. For example, widespread attitudes about organ donation, or a public reaction to a hospital audit as it has been portrayed in the media.* | No data coded to this attribute | No data coded to this attribute | No data coded to this attribute | |
| **13) Leadership** |  |  |  | |
| *The direction of a clinical team or management of a healthcare organization.* | No data coded to this attribute | No data coded to this attribute | No data coded to this attribute | |
| **14) Regulatory or Legislative Standards** |  |  |  | |
| *Statutes or principles established and enforced by an agency external to the medical profession. Regulatory or legislative standards are here distinguished from guidelines insofar as these standards are binding, often based on law or remuneration structures, and are outside the control of health organizations.* |  | - In all countries there is a law that specifies the right to protection from any form of maltreatment or humiliation of people with mental illness. All countries have official strategies for improving mental healthcare services, with commonalities around protection and improvement of mental health of their inhabitants. However, a large inconsistency between the strategies and defined action plans was reported. | **Legal:** Silence (Policy Analysis) | |

* UN Resolution. CMHCs = community mental health centres; NGOs = non-governmental organizations; FGs = focus groups; CBT = cognitive behavioural therapy
