# Supplementary material for: Process evaluation of a hybrid effectiveness-implementation, pragmatic, cluster randomised controlled trial (IMPULSE) to improve psychosocial treatment of patients with psychotic-spectrum disorders in Southeast Europe
Source: PLoS One. 2026 Feb 4;21(2):e0338408. doi: 10.1371/journal.pone.0338408 (PMC12872023; doi:10.1371/journal.pone.0338408)
Supplement: S2 Table — In-depth description of all themes interpreted from the interviews with clinicians and patients with illustrative quotes. (DOCX) [file pone.0338408.s003.docx]

**Supporting Table 3.** Interviewed patient characteristics by site and intervention acceptability-related themes with direct quotes per component construct of the Theoretical Framework of Acceptability.

| **Interviewed patient characteristics by site** | Bosnia and Herzegovina (n=8): 50% of patients were male (n=4). Patients were on average 34 years old (SD=10.17, range 22 to 47). The majority of the patient participants (n=6, 75%) had diagnosis of schizophrenic and related disorders (ICD-10 F20-29), whereas the remaining patients had diagnosis of mood affective disorders (ICD-10 F31). All patients attended all 6 intervention sessions, except one who attended 5 sessions.  Kosovo United Nations Resolution (n=8): 62.5% of patients were male (n=5). Patients were on average 48 years old (SD=7.10, range 34 to 57). All patient participants (n=8, 100%) had diagnosis of schizophrenic and related disorders (ICD-10 F20-29). All patients attended all 6 intervention sessions.  Montenegro (n=7): 29% of patients were male (n=2). Patients were on average 42 years old (SD=9.7, range 23 to 53). The majority of the patient participants (n=6, 85.7%) had diagnosis of schizophrenic and related disorders (ICD-10 F20-29), whereas the remaining patients had diagnosis of mood affective disorders (ICD-10 F31). All patients attended all 6 intervention sessions.  North Macedonia (n=8): 38% of patients were male (n=3). Patients were on average 41 years old (SD=6.71, range 33 to 53). The majority of the patient participants (n=5, 62.5%) had diagnosis of schizophrenic and related disorders (ICD-10 F20-29), whereas the remaining patients had diagnosis of mood affective disorders (ICD-10 F31). All patients attended all 6 intervention sessions.  Serbia (n=9): 78% of patients were male (n=7). Patients were on average 50 years old (SD=9.21, range 37 to 67). The majority of the patient participants (n=7, 77.8%) had diagnosis of schizophrenic and related disorders (ICD-10 F20-29,) whereas the remaining patients had diagnosis of mood affective disorders (ICD-10 F31). All patients attended all 6 intervention sessions. |
| --- | --- |
| **1. AFFECTIVE ATTITUDE** | |
| 1.1. Meeting patient's need for significance and empowerment | Many clinicians and patients reported that participating in the DIALOG+ sessions made patients feel important and appreciated.   - *"What really impressed me is when we called patients for [DIALOG+ sessions], they felt like we appreciated them... They felt important because someone cared about their needs." (CKOS4)* - *“Patients got a feeling that they are important, that we are interested in some areas that we do not talk about to such an extent, so they felt as if they were given some kind of additional attention and somehow, some significance…” (CMON5)*   A large number of patients reported that during DIALOG+ sessions they felt that their voice was heard and their needs were addressed.   - *"I felt really good, just kind of ... I don't know how to explain ... I felt like I was important and that everything I say really matters." (PBOS4)* - *"Honestly, I was ... I felt some relief, because someone next to me is planning, that is, helping me to overcome some difficulties." (PMON5)*   Moreover, patients stated that they were provided enough space and time to express their struggles.   - *"I think it had a very positive effect because I was able to express myself, I could get feedback, and decide what is the most appropriate to do and to do it, and continue with all the positive activities that follow." (PMAC2)*   Patients spoke about activities and tasks that were defined during the session, describing them as a particularly engaging and empowering element of the DIALOG+ intervention.   - *"Well [the activities] were all quite significant and useful, I really dedicated my time to them. I don't know how to single out anything now, everything was really useful and good." (PBOS7)* - *"I was encouraged to function more and more normally… I encouraged myself and went to find a job" (PMAC2)* - *"I liked that I had time to... To get to work, you understand, to get it started, to start working on the activities I defined with my doctor." (PMON5)* |
| 1.2. Sessions perceived as enjoyable | Both patients and clinicians reported experiencing DIALOG+ sessions as pleasant.   - *"I'm really pleased. I mean, I really, really learned a lot from this program, it was really nice for me, and I'm really happy." (CMON1)* - *"I enjoyed it altogether...I can really say that it was a pleasant experience." (PSER9)* - *"Well, it is more interesting now how to tell you, we only talked to the doctor, and now we talk more with the nurse, I find it more interesting." (PMAC6)*   Several participants found DIALOG+ sessions more relaxing in comparison to their usual treatment.   - *"Yes, it was a little more relaxed than usual. Somehow I didn't feel like a patient who came to see the doctor, I was more relaxed (...) coming to the doctor wasn't a nightmare for me as it used to be, more like some kind of entertainment." (PBOS3)* - *"Well, I'm telling you, I was really relaxed and it was just right for me, I didn't have any anxiety or anything and I got it out of me, someone talked to me." (PMON7)* - *"It was more relaxed. If you’re sitting next to a patient, it’s not that much formal doctor-patient atmosphere where you’re sitting behind a desk, typing something and evaluating. This was different, somewhat more casual." (CSER9)* |
| 1.3. Sessions perceived as too rigid | Both clinicians and patients expressed concerns that DIALOG+ was slightly monotonous due to the nature and pre-determined order of its content. This was perceived as burdensome with patients who preferred more spontaneity during their meetings with clinicians.   - *“It got a little boring in later sessions, I knew everything [the clinician] was going to ask me." (PBOS1)* - *“Since we followed that order, I have the impression that I could not simply talk freely about how I feel." (PSER9)* - *"Certain questions are repeated every time which in my opinion could be a potential problem, as it can lead to monotony over time (…) my dilemma remained as to the extent to which [loss of dynamism] was a consequence of real improvement and to what extent perhaps some kind of entry into the routine." (CSER6)* - *"..when conversations take place without the tablet, they are more sincere and the patients won't hesitate to talk. Because when you hold the tablet and ask them questions it is more formal” (CKOS4)*   Some clinicians reported finding DIALOG+ unnatural and repetitive. However, others reported the opposite.   - *"It used to seem to me that I'm asking the same thing over and over again. So it used to seem so repetitive and unstimulating to me." (CSER8)* - *“[DIALOG+] wasn't dull... not in a way that only they list their symptoms and I tell what I have to tell...” (CBOS3)*   *“[DIALOG+] may have been less spontaneous sometimes, but that disappeared over time." (CSER2)* |
| 1.4. Conversations perceived as too personal | Participants believed that DIALOG+ sessions were experienced as too personal by some patients with PSD. Clinicians’ impressions were that some of the patients experienced discomfort due to fear that certain intimate aspects of their lives could have been revealed and misused.   - - *"Psychotic patients perceive [DIALOG+] as… with distrust at the very least." (CMON1)*   - *"One patient was very suspicious, he said "My voice will be recorded and it will be sent to London." (CKOS1)*   Patients reported feelings of distrust and unease when they were asked about their satisfaction and thoughts regarding some of the domains covered during DIALOG+ sessions.   - *"I didn't really like certain questions... Especially the ones related to the experience of personal safety and certain intimate questions." (PSER2)* - *"Simply at first I was suspicious because I wanted to protect my private life." (PMON4)* |
| **2. BURDEN** | |
| 2.1.  Variation in views about intervention complexity and required cognitive effort | Participants talked about their experience with using the tablet during DIALOG+ sessions. Clinicians reported encountering some technical issues that occasionally disrupted their work, as well as patients’ resistant reactions to the tablet.   - *"The problem was that during longer sessions the tablet was sometimes turned off and the session had to be restarted." (CMAC2)* - *"I had some problems...In some cases the data were not saved even though the "OK" button appeared." (CKOS1)* - *"The use of the tablet occasionally was a resistance to the patients, because it required extra energy from them. Cognitive thinking, they were never in the mood for…" (CMAC5)* - *"What was a little complicated for them and perhaps a little scary is working on a device they hadn’t had a chance to use before... Maybe it confused them a little....” (CSER7)*   The view of clinicians was that lengthy or intensive DIALOG+ sessions were challenging for patients in terms of their focus and willingness to maintain the conversation.   - *"[Patients] wanted [DIALOG+] to last a little shorter... If something lasts longer than half an hour that is too much for chronic psychiatric patients. That's what we think, and so do [patients]." (CSER7)* - *“I have the impression that they simply do not have the capacity for longer sessions. Because as soon as I prolong that, I see that their concentration drops, that they become nervous…” (CMON1)*   Both patients and clinicians commented on the length of DIALOG+ sessions.   - *"They [DIALOG+ sessions] were too long... On average they lasted half an hour or more...I wish they lasted shorter if possible... If that's not possible we won't oppose." (PSER7)* - *"I think that sessions lasted just as long as they should [laughs] we got to talk about everything, and we never got tired in the sense that it was too long, so we would get exhausted." (CBOS1)*   Participants also reported on what they experienced as challenging and complex.   - *"There were some sessions after which I was completely exhausted... I needed a recovery after them." (PSER9)* - *“Well, now for some questions, how satisfied are you (…) that, you have to stop for a moment and start analyzing from 1 to 7... It's easier to answer with yes or no, you are satisfied or not." (PBOS8)* - *"It was complicated to decide on specific activities that would help things get better." (PMON5)* - *"There were ambiguous questions ... for example - how satisfied are you with the relationship with family members... It is not entirely clear whether it refers to relatives or a partner.” (CSER6)* - *"I had to simplify the questions to the patients because they cannot understand our medical language." (CKOS6)*   The view of patients was that the intervention did not appear to be complex to participate in. Some clinicians expressed the same.   - *"It was very simple, umm... Without any problems.”(PBOS1)* - *“The activities were not difficult for me, I managed to understand it, that part was simple, very precise and clear, to figure out what should be done." (PBOS8)* - *"It wasn't hard and there were no problems... I participated without any problems.” (PSER1)* - *"That structure of the application in the tablet and plus the manual we had been studying led us to be able to use that term "simplicity" in this intervention." (CMON2)* - *“Well, everything was pretty simple, this whole system really isn't complicated. It just takes willpower and I think both [the patients] and I had it." (CBOS3)* |
| 2.2. Clinicians reported feeling disempowered to help | Clinicians reported feeling limited in helping patients with life areas that they perceived beyond their professional role and power, such as job search or any other material resources.   - *“I would feel better if I would not say anything because I am not in the position to do something. For example, the lack of money, unemployment, I can’t make them happen. This means, I can't do anything here, that’s why I don’t have what to say." (CKOS4)* - *"There are situations when you cannot think of an activity that would be adequate, because some activities go beyond the domain of both the patient and me." (CSER3)*   The view of clinicians was that helping their patients to build more secure and stable economic and social situation requires support on a governmental level.   - *“It was also difficult, for example, some of the patients may have wanted to get away from their place of residence and take a short walk, whether in another city, on a mountain, on a lake, etc. but their economic situation does not allow them to do so.” (CMAC6)* - *"We could not offer them what they asked for. In non-material aspect yes, but regarding material aspect we can't." (CKOS8)* - *"For example, I have this patient who loves history, loves sports, but his social circumstances are a disaster ... so he barely has a pension, he doesn't have a computer ... so it would mean to me if I could have some kind of support to provide that man with at least some basic things (…) how can I help someone if they are troubled by a financial situation? Simply, I am a psychiatrist, obviously I would need extra help." (CSER8)* |
| 2.3. Using the patient’s booklet required too much effort | Clinicians’ view was that patients’ found the patient’s booklet difficult to use.   - *"They kept records maybe the first day and after that nothing, after the first time it was really, there was no chance that I would make them keep such a diary of obligations or something." (CMON1)* - *"Why do I have to write it down now? To record everything they said?” They say it's okay, but somehow it bothers them. This was usually the case with paranoid patients.'' (CSER2)*   Patients similarly reported that having the obligation to use the booklets as they were instructed was challenging.   - *"Filling out the patient booklet was the only thing that was difficult for me... I don't like to write by hand." (PSER5)* |
| **3. OPPORTUNITY-COST** | |
| 3.1. Intervention perceived as additional workload | Few clinicians reported that scheduling and conducting DIALOG+ sessions overlapped with their regular job duties and tasks, occasionally leading them to work overtime or use their breaks in order to meet their patients for DIALOG+ sessions.   - *"We somehow scheduled sessions so I would talk with them instead of using my break." (CBOS2)* - *"I would come a little bit earlier, before other regular outpatient examinations and psychotherapy sessions, and then we would talk. That's how. I would spend some extra time, because I have to work the whole afternoon..." (CSER2)* - *"But, I think that such an engagement required additional energy, time, engagement that exceeded the regular working hours, of course the scheduled term and should be adequately compensated with appropriate material means.'' (CMAC5)*   However, the majority of participants did not report any opportunity-costs related to their participating in the intervention |
| **4. ETHICALITY** | |
| 4.1. Intervention perceived as a good fit to existing clinical practice | In number of accounts clinicians stated that it would not be unusual for DIALOG+ to be offered in their outpatient clinics as they perceive it as similar to their regular work. This insight is important as it indicates that DIALOG+ implementation did not require complete reorganization of clinical work and fitted well in the existing clinicians’ and patients’ value system.   - *"There is now no significant difference between DIALOG+ and our regular work." (CSER1)* - *"Even before the [IMPULSE] project we held therapeutic sessions with patients, with the same focus." (CKOS4)* - *"I think that DIALOG+ sessions are suitable for my clinical practice... they require a little more time and better organization, but they are applicable." (CSER2)*   Some patients also perceived DIALOG+ as similar to the usual treatment.   - *"DIALOG+ sessions did not differ much from my regular clinical appointments... They were similar." (PSER6)*   Many patients, however, pointed out to a more detailed approach clinicians had during DIALOG+ sessions.   - *"So, in these DIALOG + meetings, you simply have advice on what and how to do, to try to change something for the better, in relation to what bothers you at that moment, what aches you, what makes you nervous. And on regular check-ups, for example, these are the essential questions "how you are, how you feel, whether there is a drop in mood, whether you may be too active or too passive, how much you like the therapy", and if I say I feel a drop in mood, a drop in activity, the doctor reacts immediately with the intensification of therapy." (PMON1)* - *"More domains are covered in DIALOG+ and in more details" (PSER9)* |
| 4.2. Compatibility of intervention with perceived “best clinical practice” | Participants expressed positive views regarding the type of care that DIALOG+ is specifically designed to deliver for patients with PSD. Clinicians pointed out the importance of keeping pace with contemporary psychiatric practices.   - *"Well this represents modernization of psychiatric methods with evidence-based medicine, I think things like that always improve service." (CBOS1)* - *“I think that in our institution, things that are new and have an active approach are valued." (CSER9)* - *"[DIALOG+] suits me because I like that a little bit of work is done with [patients], which is not just about drugs (…) because I know that for schizophrenia we only have pharmacotherapy and that's it, there are no support groups, there are no psychotherapeutic treatments, nothing, nothing." (CMON1)*   Similarly, patients reported to value the proactive, individualized and psychosocial therapeutic approach of DIALOG+.   - *"I will say this again, at least in my case, these conversations mean a lot. Conversations are more important to me than medications." (PBOS6)* - *"Well, for a simple check-up, when I come, those doctors don't listen to you, if I'm mentally ill, I'm not crazy, you know. I get it all. And a lot of doctors are like "give him a pill and let him go home", you know. And this is different here. So you call me, to come to psychiatry, we talk and first he asks me questions about the pills and also about other stuff, and that's what other doctors didn't do, you know, they just give pills and that's it, that's it." (PMON2)* - *“In my opinion, I think that, the doctor heals you more with words than with pharmacotherapy. That when he welcomes you well, and speaks well and speaks calmly, a person is healed with good words.” (PKOS1)* |
| **5. SELF-EFFICACY** | |
| 5.1. Ability to gradually develop confidence to receive and deliver the intervention | The ability of both clinicians and patients to allow themselves for time to learn to navigate through the features of DIALOG+ emerged from the participants’ accounts. Clinicians talked about the importance of taking time to familiarize patients with the new intervention. This approach potentially reduced the feelings of uncertainty and discomfort that patients experienced during their first sessions.   - *"I was quite reserved on that first meeting, less on the second, and already on the third I was completely open to dialogue.” (PMON5)* - *"It was easier the second and the third time because I didn't know what they would ask me on the first session (…) later I knew what will happen on the session." (PBOS4)* - *"Well, from the beginning, I was a bit... regardless of many years of work experience, I felt a bit uncomfortable... I questioned myself if I will be able to cope with all of [the patients] because they are all, well, so different... however, as it went on, it became completely normal to meet them, to talk with them, to address their problems..." (CBOS2)* - *"Well, in principle, the first sessions were for me to introduce them to the questions and to explain to them what those questions are for, in principle, to explain to them how to understand those questions because there are a lot of, I think, psychotic patients who perceive it as distrustful. I wanted to explain to them what they really want to know about these questions, and what they really need, what to think about when I ask them, I don't know, any of those items, and how they rate their condition from 1 to 7, how to teach themselves to assess how that condition changes, and so on." (CMON1)* - *"It became easier over time and more comfortable (…) when we explained everything, then each subsequent control was easier because they already knew what awaited them, what the questions were and what we were going to talk about." (CSER7)*   The opinion of clinicians was that DIALOG+ training and supplementary materials helped them gain confidence in their ability to properly deliver the intervention.   - *"It was useful for me to have some guidance, especially at the beginning, after the first sessions, because we didn't have the experience and we didn't know if we were conducting [DIALOG+] and everything else properly... so in any case, the guidance of your coordinators meant to me." (CSER3)* |
| 5.2. Variable level of confidence to use the intervention | Participants spoke about how confident they felt when delivering and receiving DIALOG+ intervention.   - *“I was pretty confident, without any problems." (CSER7)* - *"It was a new method that had not been tried before and wasn't confident." (CKOS2)* - *"Somehow I was more confident because I knew them from before, that made things easier for me." (CSER8)* - *"I felt completely confident." (PMON5)* - *"I didn't really feel over-confident [laughs]" (PSER1)* |
| 5.3. Institutional support | Clinicians reported varying levels of experienced support from their superiors.   - *"You know how it is here, there is no support... you get an order to do something and you do it. I don't consider that as a support." (CBOS3)* - *[DIALOG+] was considered something as a part of everyday work, so there was no special kind of support, but we simply agreed to participate in the project and that's it." (CMON6)* - *"I had a great support from my superiors and I am really grateful to the doctor who gave me her patients, I'm certainly grateful for her trust." (CSER4)* - *"I didn't have any problems due to the fact that I scheduled the patients at a time that was not actually intended for outpatient work. I had support for that and I was allowed to deliver DIALOG+ at the department." (CSER6)* - *"[The support from superiors] as far as I can remember was completely fine, we never had any problems…full support. And when it was necessary to come to Podgorica, for training or something, it was not a problem at all to come, to have a day off here at work.” (CMON2)* |
| **6. CLINICIANS’ INTERVENTION COHERENCE** | |
| 6.1. Understanding the main intervention principles | From participants’ accounts, it was interpreted that clinicians grasped the interventions main principles, such as patient involvement in treatment, positive reinforcement, solution-focused, resource-oriented and patient-centred approach.   - *"It meant to [patients] that someone noticed they were good in certain domains...it had a therapeutic effect - to realize that they were very satisfied in some areas...they were more proactive and certainly had more initiative...DIALOG+ enables the patient and the doctor to talk more, to be more spontaneous. I think that's important to patients" (CSER2)* - *“Well, [the main benefits] were achieved by the fact that we, the therapists, led the patients to come to a solution on their own." (CMON3)* - *"So I can say that we agreed more than what might happen before... how to say, the level of agreement with this [DIALOG+]was higher than the level of agreement otherwise, I guess the patient was more actively involved in the treatment now." (CBOS4)* - *“There was also patient involvement, as we responded to their requests. Initially, they submitted the request, then we examined the possibility for it to be realized and then we agreed to act." (CKOS4)* - *"To improve a particular area that was rated low, we talked together about ways the situation could be improved" (CMAC3)* - *"The focus is on the area that is problematic for the patient and he, in cooperation with the examiner, tries to find ways to improve the situation." (CMAC3)*   Clinicians reported a more therapeutic relationship being enabled through DIALOG+ - led conversations, which fostered trust, respect, closeness and empowerment.   - *"Certainly, communication in this way has helped to deepen the doctor-patient relationship." (CMAC5)* - *"We have deepened and expanded the way we communicate...our communication was excellent...I think they liked and enjoyed that special time dedicated to them...more time was devoted to them and thus the quality of communication improved" (CSER1)* - *"Patient acquiring greater trust in the clinician was the biggest change, I think it was getting better. As it went on, it got better and better..." (CBOS3)* - *“Our conversation was guided by those eleven items and then they sometimes spontaneously expanded their talk to some of their other life stories and I got to know them better" (CSER4)*   Clinicians further stated that DIALOG+ provides more time for patients to self-reflect and self-express.   - *"The program helps them understand what they need to pay attention to, then, that these symptoms don’t always have to be a disaster, the worst, but that they can change, that sometimes they get better, sometimes worse, degrade, you know, and I think that it meant a lot to them to be able to understand that” (CMON1)* - *“Trust me, I saw that they had a great need to express those feelings, emotions, worries and sorrows that they have. It helped them a lot that we had time to work more with them." (CKOS3)* - *"Patients had the opportunity to talk about their problems as much as they wanted...The clinician delivering DIALOG+ was willing to listen to them, unlike the regular check-ups where everything happens fast ..." (CSER8)*   The opinion of clinicians was that DIALOG+ was a structured and comprehensive approach.   - *"In my opinion, the main quality of this method is that in three or four simple steps you can effectively formulate the instructions on what should be done - what the patient should do, what should someone from his environment do, and what the therapist should do." (CSER6)* - *"Our conversation was more structured and had concrete formulations, it had clear guidance, and that suited me very well." (CMON1)* |
| 6.2. Understanding the intervention’s procedure | Clinicians elaborated on what took place during the sessions, which enabled us to interpret their coherence of the intervention’s procedure as described in the manual. Clinicians reported that during the sessions, patients graded their satisfaction following the DIALOG scale, selected areas to discuss in more detail, previously agreed actions were reviewed at the start of the next session, and new actions were set at the end of the session, which the patient recorded in their patient booklet.   - *“We started with a discussion about the given activities from the previous session, then evaluating those 11 domains that relate to life in general and treatment." (CMAC6)* - *“We went through the questions with the help of a tablet computer ... we agreed on the activities together... I encouraged them to come up with a solution on their own, of course in accordance with their abilities" (CSER3)* |
| 6.3. Limited intervention coherence | Some accounts pointed to limited intervention coherence by clinicians about use of tablet, selection of topics to discuss in more details, agreement and review of actions, and use of patient’s booklet. This suggests that some clinicians did not comprehend the intervention as intended.   - *"In those patients, in order to get better answers, instead of using a tablet, we passed those questions only orally in the form of a conversation and later I entered them in a tablet." (CMAC5)* - *"Depending on the patient, somewhere we worked on the activities and somewhere we stayed only on the rating, because some patients I followed were in stable remission" (CSER9)*   Some accounts by patients describing what happened during the intervention sessions also suggest limited intervention coherence by clinicians.   - *"No, I have not looked at the tablet, it was turned directly to my doctor, not towards me." (PMON7)* - *"I think that doctor chose topics that we are going to discuss." (PBOS6)* - *“To be honest, [the clinician] did not even mention [the patient’s booklet] to me." (PMON5)* |
| 6.4. Tablet perceived as useful | A large number of clinicians spoke about the various features of tablet they used during DIALOG+ sessions proved as helpful. Clinicians elaborated on how the use of tablet enabled them to effortlessly save all data and information related to their patients, and to retrieve them later on, if necessary. The tablet was perceived to be helpful in following and monitoring patients’ condition.   - *“The tablet helped me to be structured and organized, not to miss a question and most importantly to give me an objective parameter that I can show the patient as it was before, the previous session, month and how it is now.” (CMAC8)* - *"In principle, ahem, it was definitely really important to me that I could follow what they did, how they did it. As for me personally as a practitioner, the tablet really helped me." (CMON1)* - *"It is good to record on the tablet what we talked about last time, what we are talking about now, whether the situation is better or worse - in terms of assessment, evaluation and patient's general condition. So, it is useful because it records the data, it memorizes everything that was said." (PSER3)* |
| 6.5. Patient's booklet perceived as useful | Clinicians from North Macedonia, reported positive impressions regarding the use of supplementary materials to DIALOG+ sessions, such as the patient’s booklet.   - *“They regularly took the notebooks and wrote the tasks, sometimes by themselves and sometimes I wrote them the agreed tasks as a reminder.” (CMAC8)* - *"All assigned activities were recorded in the patient's notebook given to him during the first session in order to have an overview of the things he does during the day and relief in expressing himself at the next session." (CMAC6)* |
| **7. PERCEIVED EFFECTIVENESS** | |
| 7.1. Intervention perceived as effective | Participants’ accounts included observations of many intended positive effects of DIALOG+. The perceived benefits for patients were: increased self-awareness, increased productivity, improved relationships with family/friends and their clinician, improved physical and mental wellbeing, increased patient involvement in treatment, improved treatment adherence and decreased doses of pharmacotherapy. These benefits related to the patient were perceived by both patients and clinicians. The monetary reimbursement for patients’ participation in the study was also mentioned as a meaningful benefit.   - *“...DIALOG+ helped me personally that I understood some things about myself that I did not understand and did not know before.” (PMAC3)* - *"When you try everything to measure/rate, you somehow see that you are not as unhappy or miserable as you have felt or as you feel most of the time." (PMAC8)* - *"It happened that we changed the dose of [pharmaco-] therapy and thanks to the intervention of DIALOG + the number of visits became more frequent." (CMON2)* - *"Well, the most important thing was definitely the work situation because I got a job half a year ago (…) [DIALOG+] really helped me focus on the field that interests me the most." (PBOS3)* - *"I think I have a benefit after [receiving DIALOG+], so somehow I became bolder, stronger, I do not know how to say, so I experience that it ... leads to a positive mood, a positive thinking" (PMAC7)* - *“Well, like my sister says ... we haven't had an argument for such a long time [laughs] we really don't fight anymore and we are in lockdown at the moment, so we're in the house most of the time, this is very challenging. I think I'm reacting a little bit better now.” (PBOS8)* - *“There were all sorts of [benefits to DIALOG+], but mostly maybe that I applied to volunteer as a firefighter. I didn't know what to do, I didn't have a job, it's not just a financial problem, but I felt useless somehow, also that I was a burden to my brother (...) And then the doctor suggested I try at the fire department, I would never come up with that by myself. And that's good, I get some money on a monthly basis, but I'm also socializing, it's much better now." (PBOS4)* - *"These tasks have also influenced the change of patients' routine and have had a positive effect. For example, there were patients who did not have any friends, but when it came to friendship, we pushed them to be the initiators of the idea that they should hangout and chat with the others and this way, we helped them create new friendships." (CKOS4)* - *"[Patients] started taking initiative and being more responsible for their own condition." (CBOS2)*   The perceived benefits for clinicians were: improved communication skills and ability to establish a therapeutic relationship, increased insight into patient’s overall condition, improved clinical skills, and increased insight into the effects of the clinician’s work with the patient.   - - *“DIALOG+ enabled me to gain a better insight into the patient's overall condition." (CSER1)*   - *"The way I experienced patients has changed a lot for me in the sense that I realized that they can be helped more than just with pharmacotherapy, I mean only drugs. And that's really, really good because, in principle, they can integrate a lot." (CMON1)* |
| 7.2.  Intervention effectiveness seen as dependent on illness and personal characteristics | Patients expressed that those who are introspective, committed, accepting that they need help and willing to receive it, open-minded to try new things and take action to help themselves, benefit more from DIALOG+. Likewise, clinicians reported that it was easier to deliver the intervention to more talkative patients, who were open to communication and change.   - *“People who are very passive might not be aware of their problems ... I'm not sure how much [DIALOG+] would benefit them, it depends on their personality" (PSER2)* - *"Admittedly, it depends on the person, personality... it may be a little harder for someone to talk or exactly grade how big a problem is, so it might be harder for some people, but for me, who is generally more introspective and really often thinking about those things... for people like me, I think it's a great thing." (PBOS5)* - *"it went easy with talkative patients" (CSER8)* - *“Before DIALOG+, I chose patients I knew were open to communication, and to change." (CMON6)*   The opinion of clinicians was that DIALOG+ would particularly benefit younger patients, because the intervention was viewed to be more acceptable among this group. Some patients expressed similar views, linked to the use of tablet technology during the intervention. Similarly, clinicians reported hesitancy over the acceptability of DIALOG+ among older doctors, which would limit the intervention’s effectiveness.   - *“All of us who participated in the study were relatively younger doctors, and I don't know how our older colleagues who are about to retire would cope with all this... it is certainly more acceptable and closer to the younger generation... younger patients with preserved cognitive abilities would cooperate much better and I could help them much more." (CSER5)* - “*this younger generation may find the tablet more interesting in terms of these electronics that are used today...maybe it will be even more effective with them than with me in these years" (PMAC7)*   One clinician elaborated that their male patients reacted better to the intervention in comparison to their female patients.   - *"I was very surprised to notice that men reacted better [to DIALOG+], that male patients were somehow more responsible ... it seems to me that female patients stagnated more ... maybe somehow unintentionally, in outpatient examinations, we talk more with women than with male patients. it was surprising to me that men reacted nicely to DIALOG+.”*   Clinicians stated that patients’ motivation to engage in the intervention sessions was also influencing the intervention effectiveness.   - *"As soon as they were motivated to participate, then they saw some benefits for themselves, so in that sense, those who approached that conversation with more motivation and trust that this can help them, they were more proactive." (CMON4)* - *"They don't have, uh, the will to do things, I don't know ... to go out, to walk... I would constantly insist, they talked about their problems, I don't know, my leg hurts, my spine hurts... they don't feel well, they are not in the mood, not feeling like it... they were not even giving the real reasons, but they just didn't feel motivated..." (CBOS2)*   Clinicians from Kosovo UN Resolution elaborated that payment for participating in the study was a big motivator for patients to attend the intervention sessions.   - *"For example some patients participated on interviews only to take the money." (CKOS6)* - *“Payment has motivated patients a lot to come and hold sessions with iPad. Indeed, the payment has pushed them a lot to come" (CKOS1)*   Patients stated that being in remission or feeling stable, facilitated the participation in the intervention, whereas experiencing poor mental health prevented participating in the sessions as intended.   - *“Admittedly, I haven’t had a worsening of my illness in three years, since the last hospitalization. I don't know, maybe it would have been harder if I was in that condition, but now it was good, I just felt good every time." (PBOS3)* - *"Well, yes, so only my health condition contributed to the DIALOG+ session not being so sober and I was not 100% concentrated." (PMON1)*   This view was shared by clinicians as well.   - *“I worked with patients with chronic long-term psychosis accompanied by significant organic changes... I think that greatly contributed to the project itself not showing the expected results...I am sure that the effect would be greater in patients with different psychopathology" (CSER5)* - *“Well it was good with this group of mine because, I think, they were good and functional and everything... it went great with the tablet. But I think if the patient's condition is getting worse then... the tablet wouldn't make any sense." (CBOS3)* - *"It all depends again from patient to patient, some were in a better mental state so they could cooperate better, and some were in a worse state so it was harder to work with them." (CMON5)* |
| 7.3. Intervention effectiveness is dependent on the degree of familiarity between clinicians and patients | The view of some participants was that previous familiarity between the clinician and patient using the new intervention, enabled improvements in patients’ condition due to the already established therapeutic rapport.   - *"And because I had patients I knew, who were familiar to me from before, and then I could follow them better, say when we defined those activities, I knew them, I knew some family members, so I could be as specific as possible to suggest exactly what they need, because they were not some strangers from before, it was also a very important factor, so it goes without saying that it is easier for us when we are familiar with each other." (CMON2)*   However, the opinion of some clinicians from Kosovo UN Resolution was that using DIALOG+ with previously known patients limited their belief that significant improvements were possible.   - *"Clients were clients that we knew for a long time and I was not sure on how to expect this change." (CKOS2)* - *"Also, is good to have new clients for [DIALOG+] and not like the ones we have known for 20 years. And when the new client comes, everything is new for both of us and it is a better job." (CKOS5)* |
| 7.4. Limited experienced effectiveness | Several participants talked about not observing any particular effect of DIALOG+, nor any substantial difference in effectiveness between routine care and DIALOG+.   - *"I don't think anything has changed too much... because ... one year is a short period for some big shifts in my illness ... it takes many more years to make it better, to say it's good." (PBOS1)* - *"We didn't achieve the results that I expected. I don't think we've moved too much from the starting point, but on the other hand, nothing has gotten worse [laughs], which in a way seems good to me." (CSER5)* - *"I didn't notice any particular changes in the behaviour or communication patterns of my patients during the study" (CSER1)*   Additionally, the view of some clinicians was that the inflexible schedule of sessions limited treatment effectiveness.   - *"What I think is that sometimes the interval between two sessions, between two meetings is too long, and my personal opinion is that we would have achieved a better effect with this patient if we were in more frequent contact with her, even though the telephone line. Because I think that we should constantly remind certain patients of the agreements made, because if we make an interval of two months, they already forget what we agreed on, they forget that they came to us." (CMON3)* - *“Some patients needed to have sessions every two weeks, but the procedure didn't allow that... and some patients needed less frequent sessions" (CSER7)* |

Participants whose quotes are included are referred to with IDs created using the following strategy: letter **P** or **C** for patient or clinician, respectively; site where the interview took place (**BIH** for Bosnia and Herzegovina**, KOS** for Kosovo UN Resolution**, MAC** for North Macedonia**, MON** for Montenegro**, SER** for Serbia); consecutive number of interviewees; letter **R** for interviewer.
