# Supplementary material for: Process evaluation of a hybrid effectiveness-implementation, pragmatic, cluster randomised controlled trial (IMPULSE) to improve psychosocial treatment of patients with psychotic-spectrum disorders in Southeast Europe
Source: PLoS One. 2026 Feb 4;21(2):e0338408. doi: 10.1371/journal.pone.0338408 (PMC12872023; doi:10.1371/journal.pone.0338408)
Supplement: S3 Table — Additional in-depth findings related to each investigated domain of intervention fidelity. (DOCX) [file pone.0338408.s004.docx]

**Supporting Table 4.** Intervention fidelity findings related to training, delivery, receipt, enactment and differentiation (based on Bellg et al., 2004 and Carroll et al., 2007).

| **Training of clinicians** | All clinicians (n=41, 100%) attended three DIALOG+ training sessions: 1) Core training before starting to deliver the intervention (February - March 2019), 2) First top-up training once the clinician completed the first DIALOG+ session with all of their allocated patients (April-May 2019), and 3) Second top-up training before clinicians started delivering the fourth intervention sessions (August-November 2019) due to the long three month interval scheduled between the third and fourth sessions. All training sessions were delivered by unblinded members of the local research teams.  The core training lasted between 1 and 3 hours in all countries, except in Kosovo* where it lasted more than 3 hours, and it was organized both individually and as a group training. In North Macedonia, Bosnia & Herzegovina, and Montenegro the first top-up training lasted up to 1 hour, in Serbia it took between 1 to 3 hours and in Kosovo* it lasted more than 3 hours. This top-up training was organized both individually and as a group. The second top-up training lasted up to 1 hour in North Macedonia, Bosnia & Herzegovina, and Montenegro, and between 1 and 3 hours in Serbia and Kosovo*. Most countries organized this training with each clinician individually, while in Kosovo* it was organized as a group training.  At the first top-up training session, researchers collected data regarding the completion of activities related to preparation and training for intervention delivery. They reported that each clinician from the intervention arm received a tablet needed for the DIALOG+ sessions during the training (n=41, 100%). All tablets were set up with an app in the local languages (n=41, 100%). Each clinician received the DIALOG+ manual, clinician’s pack and implementation strategy (n=41, 100%). Researchers from Kosovo*, Montenegro, Serbia and North Macedonia reported that each clinician from the intervention arm read the manual (n=33, 80.49%). Researchers from Bosnia and Herzegovina reported that some of the clinicians from their site did not read the manual due to lack of time. Researchers from Serbia reported that not every clinician who attended the training, read the manual thoroughly. However, they sufficiently familiarized themselves with it. All clinicians (n=41, 100%) in the intervention arm from all research sites saw the presentation and video demonstrations, important elements of the DIALOG+ training. Moreover, all clinicians (n=41, 100%) practiced role-play vignettes as well as DIALOG+ simulation using the app and tablet.  In the qualitative interviews with clinicians from the intervention arm, their opinion was that the training and supplementary materials they received prior to DIALOG+ implementation helped them gain confidence in their ability to properly deliver the intervention.   - *"Everything was fine, the training explained, let's say, 90% of things, so there were no problems with using it. And I could always call one of you if I got stuck, so that, too, was very good and helpful." (CBOS1)* - *"The manual we received contributed a lot to us, it was very reliable in the preparation of this intervention. Only that manual I think is one of the essential factors. It should be studied well, because again it can't be just one reading or another, there are a lot of things and it's just... And literature, it all needs to be studied well to get into this project and into the session, and to get better results." (CMON2)*   Additionally, from the data collected by researchers at the first top-up training session, researchers from Montenegro reported that clinicians found video demonstrations useful because they provided a good overview of how a DIALOG+ sessions should look like and how to develop conversations during the sessions. |
| --- | --- |
| **Delivery of intervention** | 221/236 (93.64%) patients received at least one DIALOG+ session and 189/236 (80.08%) received all six sessions. The below table summarizes the occurrence and attendance of all intervention sessions.   \| *Number of intervention sessions* \| **Session 1** \| **Session 2** \| **Session 3** \| **Session 4** \| **Session 5** \| **Session 6** \| **Total** \| \| --- \| --- \| --- \| --- \| --- \| --- \| --- \| --- \| \| **Intended** \| 236 \| 236 \| 236 \| 236 \| 236 \| 236 \| **1416** \| \| **Delivered** \| 221 \| 211 \| 209 \| 206 \| 200 \| 194 \| **1241** \| \| **% delivered/intended** \| 93.64% \| 93.64% \| 88.56% \| 87.29% \| 84.75% \| 82.20% \| **87.64%** \| \| **Number (%) of patients attending 1-6 sessions in total** \| 10 (4.24) \| 2 (0.85) \| 2 (0.85) \| 3 (1.27) \| 15 (6.36) \| 189 (80.08) \| **236 (100)** \|   8.3% (n=103) of all the delivered DIALOG+ sessions were conducted over a telephone call, and 2.18% (n=27) were conducted over a video call. The sessions conducted virtually or over the phone were the fifth and sixth sessions that occurred during the pandemic period. Importantly, 65.46% (n=127) of all 6^th^ DIALOG+ sessions delivered in the trial were provided remotely.  At the first top-up training, researchers further collected data from each clinician (n=41) in the intervention group if they experienced any problems with the delivery of the intervention and participating in the study. Nine clinicians (21.95%) reported experiencing problems. These included technical difficulties with the app and tablet, with scheduling sessions’ appointments and communication issues. The majority of the expressed problems were reported as successfully resolved through additional support from researchers and patients’ relatives, allowing more time for patients to open up and get acquainted with the intervention and by re-entering data in the app. The only problem reported as not resolved at the time of the survey was communication issues between one clinician and the research team, further attempts were made to improve the communication.  The below table presents the frequency of action items agreed during the DIALOG+ sessions.   \| **Number of action items per session** \| **Number of sessions (%)** \| \| \| --- \| --- \| --- \| \| 0 action items per session \| 9 \| (0.73) \| \| 1 to 3 action items per session \| 1045 \| (84.21) \| \| 4 to 6 action items per session \| 173 \| (13.94) \| \| 7 to 12 action items per session \| 14 \| (1.13) \| \| **TOTAL sessions** \| **1241** \| **(100.00)** \|   Examples of patient-led actions set during the DIALOG+ sessions: “Patient to call his friend at least 3 times per week”; “Talk to her husband to allow her to see children more often”; “Write a letter to his brother”; “Go out with a friend for a morning coffee twice per week for about an hour”; “The patient will walk for one hour every night”; “Try to reduce consumption of bread and pastries”; “Riding a bike or running in the morning hours”; “Patient will arrange a meeting with a gynecologist”; “The patient will schedule a consultation with the psychiatrist”; “Look for open vacancies in newspapers”; “The patient will go to the employment office in order to seek jobs”; “Submit request for receiving assisted financial help”; “Take her daughter out to playground this upcoming week”; “Play chess with his nephew three times a week”; “Patient will plant flowers in the garden”.  Examples of clinician-led actions set during the DIALOG+ sessions: “The clinician will coach the patient on finding employment”; “Show care toward patient by bringing a glass of water to sessions”; “Clinician will make a proper nutrition plan by the next meeting”; “Clinician will help him to write CV”; “The clinician will visit the patient's family in order to discuss relations”; “Talk to family so they understand her condition better”.  Examples of other-led actions set during the DIALOG+ sessions: “daughter will watch movies with her 3 times a week”; “Mother will remind the patient about the medication”; “family will organize playing social games 2 times a week”; “mother and sister will help her to write CV”; “Her husband and daughter to help her with everyday obligations around the housekeeping”.  Each recording obtained from the intervention sessions was scored against the DIALOG+ Adherence Scale (DAS). The mean score for the DAS subscale ‘Quality of interaction’ was the highest (2.67/3). The adherence to the 4-step procedure was moderate (6.9/9), whereas the adherence to the initial DIALOG scale and review of ratings was the lowest (4.38/7). All clinicians in this sample delivered items ‘Satisfaction - DIALOG Scale’, ‘Step 1 – Understanding (explore)’, ‘Step 3 - Exploring options (patient)’; and ‘Step 4 - Agreeing on actions’. Only two clinicians delivered item ‘Comparison’ (Table 6).  High-scoring items from the DAS (mean score ≥ 0.90) included: *‘Satisfaction – DIALOG Scale’*, showing that most or all areas on the DIALOG Scale were rated; *‘Number of areas’*, signifying that the optimal amount of areas were selected to be discussed; *‘Step 1 - Understanding (explore)’*, showing that clinicians explored with the patient their rating of the chosen area; *‘Step 3 - Exploring options (patient)’*, indicating that clinicians encouraged the patient to consider any actions that could be taken by the patient themselves; *‘Step 4 - Agreeing on actions’*, showing that actions were set to be taken in the chosen area; *‘Quality of interaction - positive regard’*, demonstrating that clinicians expressed considerable and consistent positive regard towards the patient; and *‘Quality of interaction - patient involvement in 4-step approach’*, showing that the patient was actively involved in the discussion, and at minimum was asked for their view and/or agreement during the 4-step approach. One low-scoring item from the DAS (mean score ≤ 0.25) was identified: ‘comparison’ indicating that few clinicians offered to compare the current session’s ratings with those of a previous session after all areas were rated. |
| **Receipt of intervention** | Three intervention receipt-related themes that were interpreted from the end-of-trial qualitative interviews with patients and clinicians allocated to the intervention arm through framework analysis:  *Variable views about the level of patients’ understanding of the intervention procedure*  Some clinicians expressed uncertainty whether their patients understood what DIALOG+ was about. Similarly, some patients could not recollect what took place during the intervention.   - *"I'm not sure that the patients properly understood what it was all about.” (CSER5)* - *“The difficulty was probably due to the nature of the patients' illness, that they probably didn't understand all the questions, so they gave the wrong answers because they misunderstood” (CKOS4)* - *"The tablet ... with the tablet ... the doctor used it when she asked me about the questions and to see some things, I do not know what exactly, how, what, about the assessment of something, I do not know and so …" (PMAC5)*   Others expressed a differing opinion.   - *"[DIALOG+] was pretty easy. I think it was easy for both me and them to adapt, the application is quite, let's say, internet-friendly... patients immediately figured out how to do it... the instructions from the application itself were pretty clear." (CSER9)* - *“Patients understood the purpose, they understood the program and, they simply saw it as a good way to solve certain personal problems." (CMON3)*   Thus, clinicians felt that patients were able to easily participate in the DIALOG+ sessions, except when cognitive impairments were perceived, which were observed to have limited patients’ understanding of the intervention. Many accounts from patients describing what happened during the intervention sessions, support the clinicians’ views that patients were able to understand DIALOG+.   - *"Well, I would go into the office and then we would, then we would start right away with, with the tablet, so she would ask me how satisfied I am with my mental health, with the environment, with my family, and I would have, uh, a grade of 1 to 7, that is from "Not at all satisfied" to "Satisfied", erm, as far as activities are concerned, they would come into consideration if the grade was for example 5, and below. Um, then we would usually agree on what I would do, as an individual, to be better, and so on at every other meeting." (PMON5)*   *Comprehension of intended main intervention principles*  From participants’ accounts, it was interpreted that patients were aware that their point of view was valued and respected during the intervention sessions. We also deduced that patients were able to recognize their active role during the DIALOG+ sessions and that the intervention works through providing an opportunity for therapeutic self-expression and self-reflection on a wide number of areas of wellbeing.   - *"Somehow what I say was more respected now, not in the sense that it is not respected otherwise... but I had more time and space to express myself and then I had bigger influence on the way the conversation goes. On the other hand, I felt more equal than in situations when only yes/no questions are asked" (PBOS7)* - *"Well we talked more about everything, and, I was given time to say what I have, I also talked about how I behave, how I feel, what I need, we also talk during check-ups but this is much more, these meetings that we had, a lot more thoughts got released and the doctor advised me a lot, and I said some thoughts that I had not expressed before." (PMON6)* - *"I was able to better understand my physical and mental condition [with DIALOG+]" (PSER5)* - *“It was easier for them to get to the heart of the problem, and I have the impression that they found it easier to see the reasons for the low scores on their assessment" (CSER3)*   Additionally, we interpreted that patients were able to understand that the intervention adopts a solution-focused approach.   - *“[with DIALOG+] I was able to go into more detail and I could start solving things." (PBOS8)* - *“In [DIALOG+] sessions we talk about the future, how to move forward." (PMON2)* - *"it was effective, focused directly to the core of the given problems or difficulties that vary over time and to finding a crucial solution...how to go on" (PMAC2)* - *“...then I come to the realization that what was difficult for me and what, I don't know, represented a huge problem and burden... is solved or simplified by the next session in a way that I can say, express, do something... then at some point it became hard for me to make an elephant out of it all when it really comes to being... a butterfly." (PBOS2)*   Furthermore, from the data, we interpreted that patients recognized the value of DIALOG+ that relates to adding structure to clinician-patient meetings to ensure that the session is more comprehensive and easier.   - *“[with DIALOG+] there is a certain method and order and everything is much easier then…” (PSER9)* - *"it somehow includes all segments, it literally has a question from every segment of life... while routine control sessions basically boil down to how you are, do you feel good, do you have symptoms or suicidal thoughts, how do you sleep, how do you eat... these are issues that are involved in routine sessions, while the tablet, let's say, expands, complements all of that" (PBOS7)*   This indicated that patients understood the intervention’s main principles (i.e. its mechanisms of action).  *Patients’ booklet perceived as useful*  Patients elaborated on different ways in which they used their booklet, either as a mean that helped them memorize and organize tasks they defined during DIALOG+ sessions, or as a tool they used in order to reflect on their condition and symptoms. The booklet was designed in order to help patients keep track of their activities and tasks they set up with their clinicians during DIALOG+ sessions.   - - *"Yes, I used it, I would often read it and analyze it as well." (PBOS8)*   - *"I had that diary that we got, so I wrote down some things that were important to me - about myself, certain events, problems and so on. I also wrote down the doctor's advices there, and then I could occasionally remind myself." (PSER1)*   - *“I wrote down everything we talked about and my aspirations...I read that every day." (PSER2)*   - *“I wrote the tasks in the booklet, I'm still carrying it with me all the time." (PSER9)*   - *"Well, yes, I have that booklet and she [the clinician] told me to write there until the next session. I was writing with a date - this day I was making, for example cakes, another day lunch, certainly useful" (PMAC6)*   This indicates that use of the booklet during the trial was adjusted by each patient according to their specific needs at the time. |
| **Enactment of intervention** | Enactment-related themes that were interpreted from the end-of-trial qualitative interviews with patients and clinicians allocated to the intervention arm through framework analysis:  *Agreed actions were difficult to implement*  Clinicians and patients reported that patients were not able to complete the agreed actions/tasks between sessions.   - *"I didn't complete a lot of homework tasks, I had a lot of problems with that" (PSER8)* - *“Well, sometimes patients would promise to do some things, but did not, they neglected them." (CMON3)* - *"To be honest like I am now, [completing activities] was not simple" (PMAC7)*   This indicates that some patients did not comply with the tasks agreed to be conducted in between the sessions.  *External factors limited enactment*  The opinion of participants was that restricted access to resources and support and other responsibilities hindered patients in the process of completing the agreed actions.   - *"For example, I have this patient who loves history, loves sports, but his social circumstances are a disaster ... so he barely has a pension, he doesn't have a computer ... (CSER8)* - *"Something else that was difficult was that we could not realize the action articles as we wanted. Reason number one were the conditions here. If our social conditions were better DIALOG+ would be so much more successful." (CKOS6)* - *"I liked everything except some homework that I failed to complete. The doctor and I were helpless in those situations, something we wanted didn't turn out the way we agreed because a third person was involved, my ex-wife... so I failed to accomplish some things that doctor and I agreed on." (PSER3)* - *"I haven't enrolled in any course, neither for free, nor commercially... because I'm telling you, my two sons go to school, it's a little harder with this little one, we have to help him with studying a lot (...) So I generally don't have much time for courses, activities and similar things." (PBOS1)*   This suggests that the difficulties experienced with completing actions could often be outside of the patients’ control.  *Internal factors limited enactment*  In addition to lack of outside resources, it was participants’ view that lack of patients’ motivation and willingness to proactively take up the agreed actions and negative symptoms were a hindrance to completing the tasks in between sessions.   - *"They don't have, uh, the will to do things... to go out, to walk... I would constantly insist, they talked about their problems, (…) they were not even giving the real reasons, but they just didn't feel motivated..." (CBOS2)* - *“They just weren't persistent even in some seemingly simple things and didn't manage to complete the actions until the next session ... somehow they didn't try hard enough. They tried but they gave up very quickly. It used to be a problem for them even to go for a walk every day, which seemed to be a simple task during the summer." (CSER5)* - *“It was rare for a patient to take the initiative to perform the assigned activities independently and responsibly." (CMAC5)* - *"I was not able to solve these problems immediately, I kind of postponed everything" (PSER1)* - *"...but I did not always complete all of the task, I was simply in the phase where I could not do much." (PBOS6)* - *"Well, given the mental fatigue as a patient... I was tired because I cannot concentrate much on the health or life problems, I could not concentrate much, it was hard" (PMAC5)*   *Agreed actions were enacted in daily life*  Participants also commented that patients completed set actions in between sessions.   - *"I managed to complete all the agreed activities" (PSER4)* - *“I was looking at the brochure and performing the tasks [the doctor] would write in the brochure. I have successfully completed every task of the doctor." (PMON4)* - “*We usually agreed on simple tasks, so they would generally complete them." (CBOS3)* - *"Most of the [patients] tried to accomplish what we set out to do." (CKOS6)*   Some patients and clinicians reported that patients were successful in completing activities.  *Well-tailored actions and a proactive attitude facilitated the implementation of actions in daily life*  Patients described that it was feasible for them to complete actions which they agreed with their clinician during the intervention sessions. We interpreted that having a proactive attitude and setting actions tailored to the patient’s specific needs and abilities, facilitated the implementation of actions in patient’s daily life.   - *"I think DIALOG+ helped me the most when it comes to getting a job...we agreed on the task of printing those ads with phone numbers and posting them. I did that...it didn’t take long and I got my first job." (PSER9)* - *"Then we talk about - what to do? I said [to the clinician] I will cook. Then I started to cook more often, to pass the time, to be better, to have some...  to have responsibilities"(PMAC6)* - *"Well, yes, I was able to complete a big part of it ...so, it is easy to work on the things that are completely related to me, that depend entirely on me, and that was the easy" (PMAC8)* - *“I was capable [to complete the actions] because I decided on some activities that were simple for me. And if I couldn’t do an activity, I would tell [the clinician] that I can't, for example, to run.” (PKOS4)* - *"One of my patients who had a problem whether to go to a course, to one of the courses or not, I said "okay, let's make it a part of our activity so we can see what happens", and she really finished it, and now she can work as a freelancer and that's a great thing." (CMON6)* - *"We did not make any grandiose plans that we knew they would not be able to realize. We took small steps towards the goal." (CSER4)* |
| **Intervention differentiation** | Of all delivered control sessions, 8.38% (n=111) were conducted over a telephone call, which were the fifth and sixth sessions during the pandemic period. Notably, 47.83% (n=99) of all 6^th^ control sessions delivered in the trial were provided remotely.  Each recording obtained from the control sessions was scored against the DIALOG+ Adherence Scale (DAS). Control clinicians scored less than half the possible total score on the DAS subscale ‘Quality of interaction’ (1.25/3), compared with 2.67/3 for intervention clinicians. Use of the 4-step procedure, the initial DIALOG scale and review of ratings were low (1.5/9 and 0.75/7, respectively), whereas intervention clinicians scored 6.9/9 on the 4-step procedure subscale and 4.38/7 on the DIALOG procedure (Select & Review) subscale. |

*By United Nations Resolution
